# Rare Variants of Dermatofibrosarcoma Protuberans: Clinical, Histologic, and Molecular Features and Diagnostic Pitfalls

**DOI:** 10.3390/dermatopathology10010008

**Published:** 2023-01-29

**Authors:** Celestine M. Trinidad, Sintawat Wangsiricharoen, Victor G. Prieto, Phyu P. Aung

**Affiliations:** 1 Department of Anatomic Pathology, Benavides Cancer Institute, University of Santo Tomas Hospital, Manila 1008, Philippines; 2 Dermatopathology Section, Department of Pathology, The University of Texas MD Anderson Cancer Center, Houston, TX 77030, USA

**Keywords:** DFSP, rare subtypes, histology, clinical and molecular

## Abstract

Dermatofibrosarcoma protuberans (DFSP) is a dermal malignant mesenchymal tumor. Most variants are associated with a high risk of local recurrence and a low risk of metastasis. The classic histomorphology of this tumor is made up of uniform, spindle-shaped cells, arranged in a storiform pattern. Tumor cells characteristically infiltrate the underlying subcutis in a honeycomb pattern. Less common variants of DFSP have been identified: myxoid, pigmented, myoid, granular cell, sclerosing, atrophic, and fibrosarcomatous. Only the fibrosarcomatous variant has been shown to differ significantly from classic DFSP in terms of clinical outcome; fibrosarcomatous DFSP has been shown to be associated with a greater risk of local recurrence and metastatic potential than classic DFSP. However, the other variants may pose diagnostic difficulty as they resemble other types of spindle cell neoplasms, especially in small biopsy specimens. This article reviews the clinical, histologic, and molecular features of DFSP variants, as well as possible pitfalls in their diagnosis and how to resolve them.

## 1. Introduction

Dermatofibrosarcoma protuberans (DFSP) is a mesenchymal neoplasm of low to intermediate grade, with fibroblastic or myofibroblastic differentiation. DFSP is most commonly seen on the trunk and the proximal extremities of young to middle-aged adults. DFSP is locally aggressive and has a high local recurrence rate, but its metastatic potential is low [1,2].

**Figure 1:**
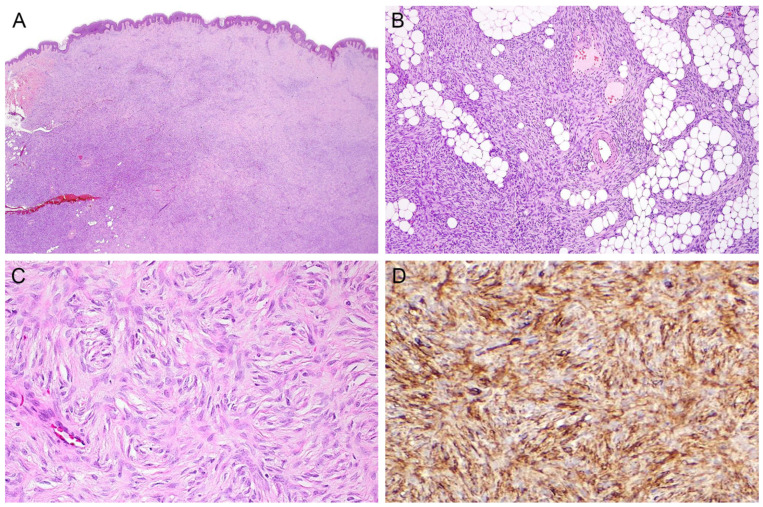
DFSP with classic histomorphology: dermal-based tumor with uniform, spindle-shaped cells ((**A**); magnification ×40) arranged in a predominantly storiform pattern ((**B**); magnification ×100 and (**C**); magnification ×200). These cells are primarily based in the dermis, with infiltration of the subcutis in a characteristic diffuse honeycomb fashion (**B**). In immunohistochemistry, DFSP shows strong and diffuse positive staining for CD34 ((**D**); magnification ×200).

Clinically, DFSP usually presents as an indurated tumor nodule that protrudes above the skin surface (hence, its “protuberans” designation) [3]. The classic histomorphology of this neoplasm is uniform, spindle-shaped cells (Figure 1A), arranged in a predominantly storiform pattern (Figure 1B,C). These cells are primarily based in the dermis, with infiltration of the subcutis in a characteristic diffuse honeycomb fashion [1] (Figure 1B).

In immunohistochemistry, DFSP shows strong and diffuse positive staining for CD34 (Figure 1D) while being negative for factor XIIIA, desmin, D2-40, or CD163 (markers more commonly seen in dermatofibroma). Cytogenetically, this neoplasm is characterized by supernumerary ring chromosomes containing a centromere from chromosome 22 and material from both chromosome 22 and chromosome 17. Another alteration, unbalanced t(17;22)(q21.3;q13.1), is more common in pediatric cases. Both aberrations bring the platelet-derived growth factor subunit B gene (*PDGFB*) under the control of the collagen-type I alpha 1 chain gene (*COL1A1*) promoter [1]. *COL1A1-PDGFB* rearrangements can be detected through molecular testing, such as fluorescence in situ hybridization assays and multiplex reverse transcription polymerase chain reaction [4]. *PDGFB* RNA chromogenic in situ hybridization has also demonstrated its utility as a surrogate marker of *PDGFB* rearrangement [5].

Less common histologic variants of DFSP have been identified, namely, myxoid, pigmented, myoid, granular cell, sclerosing, atrophic, and fibrosarcomatous variants. Only the fibrosarcomatous variant has been shown to differ significantly in clinical outcome from classic DFSP. However, the other variants may pose diagnostic difficulty, as these may resemble other types of spindle cell neoplasms, especially in small biopsy specimens. The correct diagnosis of DFSP is essential, as these other similar-appearing neoplasms may have different clinical behavior and treatment options.

This article reviews the clinical and histologic features of the rare DFSP variants, as well as their differential diagnosis, possible pitfalls in diagnosis, and immunohistochemical and molecular features that may help resolve difficulties in the diagnosis of these variants.

## 2. Rare Variants of DFSP

### 2.1. Myxoid

**Figure 2:**
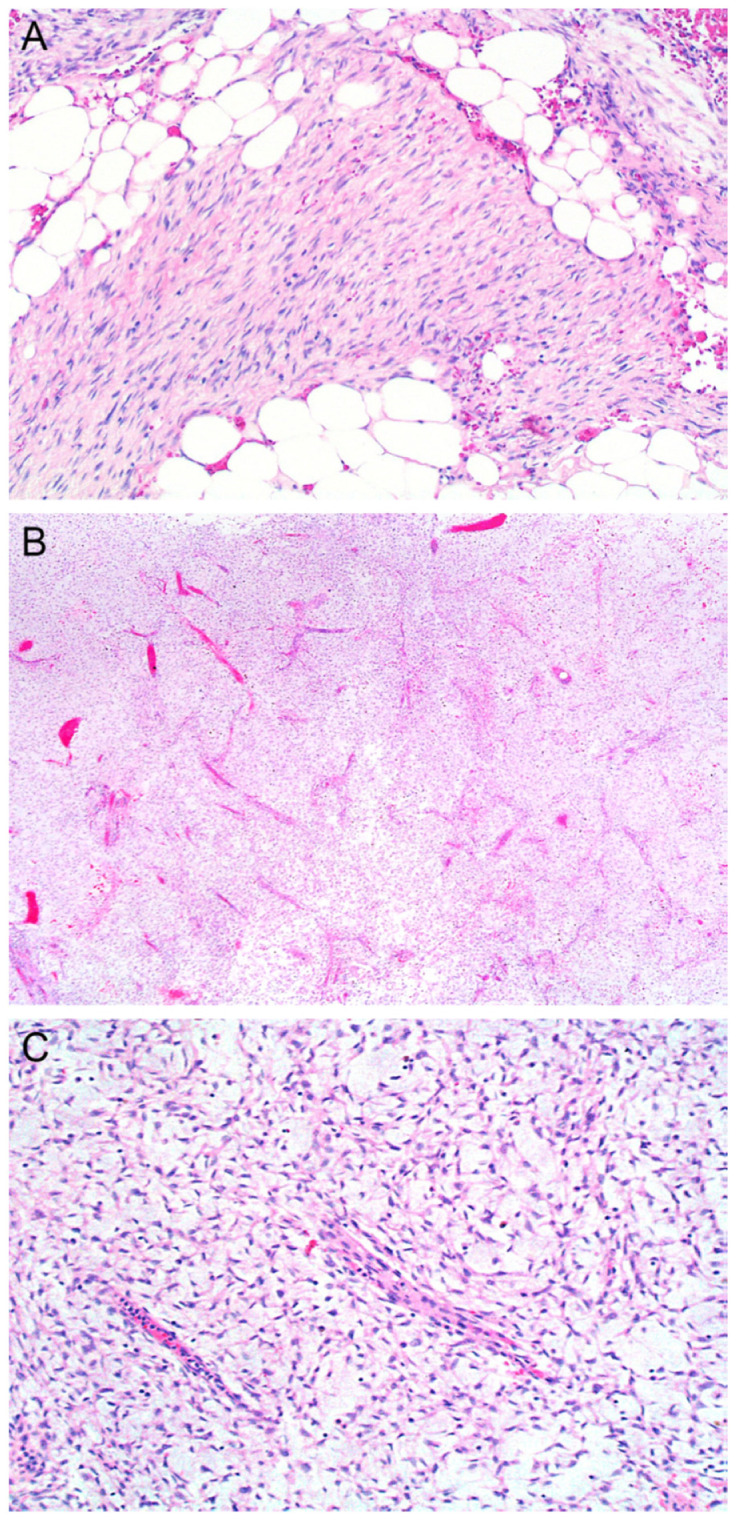
Myxoid DFSP. In this variant, characteristic storiform growth of DFSP is often lost, and the blood vessels are more prominent. However, honeycomb pattern of infiltration into subcutaneous fat can still be seen ((**A**); magnification ×100 and (**B**); magnification ×40). Nuclei are plump, wavy, and uniform with focal or diffuse myxoid change ((**C**); magnification ×100).

Myxoid DFSP was first described by Frierson and Cooper in 1983 [6]. It shows multinodular growth, abundant pale myxoid stroma, frequently branching small thin-walled vessels, and scattered mast cells. In this variant, characteristic storiform growth of DFSP is often lost, and the blood vessels are more prominent (Figure 2A,B). However, a honeycomb pattern of infiltration into subcutaneous fat and diffuse expression (Figure 2A) with CD34 can still be seen. Nuclei of the tumor cells are plump, wavy, and uniform (Figure 2C). For DFSP to be classified as myxoid, areas of myxoid change should account for more than 50% of the tumor [1] (Figure 2C).

Prominent myxoid stromal changes are observed in many benign and malignant mesenchymal neoplasms, which may be mistaken for myxoid DFSP, especially in small biopsy specimens. Neoplasms that may resemble myxoid DFSP include cutaneous myxoma, myxofibrosarcoma, low-grade fibromyxoid sarcoma, myxoid liposarcoma, superficial acral fibromyxoma, solitary fibrous tumor, and monophasic synovial sarcoma.

Unlike DFSP, cutaneous myxoma is well circumscribed and does not infiltrate the underlying subcutis. In addition, cutaneous myxoma is negative for CD34 but positive for alpha-smooth muscle actin.

Myxofibrosarcoma, even when low grade, still shows greater cytologic atypia than DFSP, with cells having enlarged and hyperchromatic nuclei. Features present in myxofibrosarcoma but not in DFSP include pseudolipoblasts, neoplastic fibroblastic cells with cytoplasmic mucin, and thin, curvilinear blood vessels [2].

Like DFSP, low-grade fibromyxoid sarcoma may present superficially, with involvement of the dermis and subcutis [7]. However, in contrast to myxoid DFSP, low-grade fibromyxoid sarcoma presents as a well-circumscribed neoplasm with pushing rather than infiltrative borders. These tumors are positive for EMA and also show strong, diffuse cytoplasmic expression of MUC4.

Myxoid liposarcoma is usually seen in the deep soft tissues of the extremities, most often the thigh, and only rarely in the subcutis, unlike DFSP. Tumor cells of myxoid liposarcoma may be associated with small lipoblasts; however, these are not always present. Myxoid liposarcoma is negative for CD34. Demonstration of *DDIT3* rearrangement (*FUS-DDIT3* or *EWSR1-DDIT3* fusion genes) is also helpful in ruling out myxoid liposarcoma [8].

Superficial acral fibromyxoma and solitary fibrous tumors are both CD34-positive tumors that may present with prominent myxoid areas and, thus, may be confused with myxoid DFSP [9,10,11]. Unlike DFSP, superficial acral fibromyxoma is mostly restricted to acral sites, predominantly on the periungual region of the digits. In contrast to DFSP, solitary fibrous tumors are well circumscribed and are associated with a branching hemangiopericytoma-like vascular pattern. Positive STAT6 staining in immunohistochemistry and demonstration of *NAB2-STAT6* gene fusion on molecular testing can help distinguish solitary fibrous tumors from DFSP [8].

Monophasic synovial sarcoma may also show prominent myxoid changes [12]. However, like myxoid liposarcoma, these also arise from the deep soft tissue of the extremities. These are CD34 negative and instead express EMA and cytokeratins, though this may be focal. TLE1 and SS18-SSX fusion-specific immunohistochemistry, as well as demonstration of SS18-SSX1/2/4 gene fusion, would also strengthen the diagnosis. 

### 2.2. Pigmented

**Figure 3:**
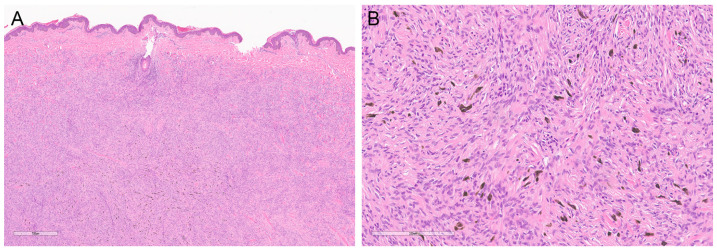
Pigmented DFSP (Bednar tumor). Occasionally, DFSP can show areas of melanocytes containing melanin pigments ((**A**); magnification ×40 and (**B**); magnification ×200). The tumor otherwise shows characteristic features of DFSP. This feature has no prognostic importance.

Pigmented DFSP is also known as Bednar tumor, as first described in 1957 by Bednar, who initially called it “storiform neurofibromas of the skin” [13]. Pigmented DFSP is associated with numerous dendritic cells containing melanin pigment [1,14] (Figure 3A,B). These pigmented cells express S100 protein (and, thus, are consistent with melanocytes) (see also next paragraph). Thus, included in the differential diagnosis of pigmented DFSP are melanocytic neoplasms, including nevi and melanoma, and a malignant melanotic nerve sheath tumor.

Immunohistochemistry in cases of pigmented DFSP highlights two populations of cells: pigmented cells that stain positive for S100 and spindle-shaped cells that are negative for S100 and positive for CD34 [15,16]. These findings are in contrast with the findings in melanocytic neoplasms, which are diffusely positive for S100 in both pigmented and nonpigmented cells. Melanocytic neoplasms are also positive for other melanocytic markers, such as Melan-A and HMB-45. Melanomas also show a greater degree of cytologic atypia than pigmented DFSP and frequent, sometimes atypical, mitoses.

In distinguishing pigmented DFSP from malignant melanotic nerve sheath tumor, location is also important, as malignant melanotic nerve sheath tumors predominantly originate from the spinal or autonomic nerves and are often seen near the midline [8]. In less pigmented areas, the tumor cells of malignant melanotic nerve sheath tumors have an eosinophilic to amphophilic cytoplasm; round to ovoid nuclei, often with nuclear grooves and pseudoinclusions; and usually small nucleoli. Co-expression of S100, SOX10, and melanocytic markers is also seen in these tumors.

### 2.3. Myoid

**Figure 4:**
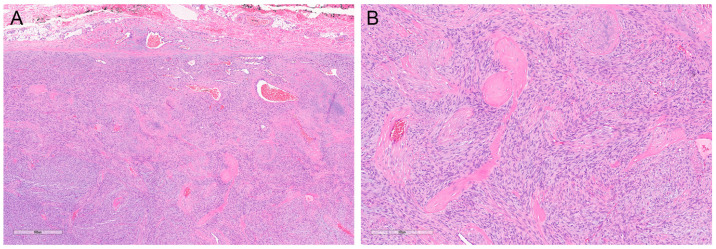
Myoid DFSP. Stromal vessels can have myointimal hyperplasia resulting in myoid nodules ((**A**); magnification ×40), which is not a usual feature in conventional DFSP—more common in DFSP with fibrosarcomatous transformation. The cell myoid nodules have more abundant bright eosinophilic cytoplasm with cigar-shaped nuclei—catachrestic of smooth muscle differentiation with SMA expression ((**B**); magnification ×100). They are positive for SMA.

Myoid DFSP was first described by Calonje and Fletcher in 1996 [17]. It is composed of nodules of spindle-shaped cells with plump nuclei and eosinophilic cytoplasm. These nodules are often perivascular, with well-defined cytoplasmic margins, and associated with stromal hyalinization [18,19] (Figure 4A,B).

Unlike classic DFSP, in myoid DFSP, the myoid area shows positive staining for alpha smooth muscle actin, and weak or negative expression of CD34, which may be a pitfall in its diagnosis. Myoid DFSP has also been reported to be associated with fibrosarcomatous areas [17]; thus, adequate sampling of the specimen is essential.

Important entities to consider in the differential diagnosis for this variant include cutaneous smooth muscle neoplasms, such as cutaneous leiomyoma and leiomyosarcoma. Leiomyosarcomas show greater cytologic atypia than myoid DFSP, including nuclear enlargement and hyperchromasia, and are more mitotically active. Smooth muscle neoplasms are positive for desmin, while myoid DFSP is negative. In difficult cases, in distinguishing myoid DFSP from these other entities, the detection of the characteristic chromosomal translocation of DFSP (*COL1A1::PDGFB* fusion) by fluorescence in situ hybridization may be helpful.

### 2.4. Atrophic

Atrophic DFSP was first described in 1985 by Lambert and colleagues [20], who termed it a “morpheaform” variant of DFSP because of its depressed and plaque-like clinical appearance, which contrasts with the protuberant nodule, typical of classic DFSP. Clinically, atrophic DFSP may be mistaken for morphea, morpheaform basal cell carcinoma, localized scleroderma, lupus, panniculitis, or hyperpigmentation [21,22].

Histologically, atrophic DFSP often shows spindle cells arranged in parallel fascicles. The usual storiform pattern of classic DFSP is not as prominent; it may be focal. Infiltration of the underlying subcutis is often seen. Because of this histomorphology, the differential diagnosis of atrophic DFSP includes other fascicular spindle cell neoplasms, including neurofibroma, dermatomyofibroma, and plaque-like CD34-positive dermal fibroma.

Neurofibroma is usually well circumscribed, but the diffuse variant of this tumor also shows infiltration of the dermis and subcutis and, thus, it may be mistaken for DFSP. In cases of neurofibroma, immunohistochemistry for S100 and SOX10 shows positive staining in some of the cells. In contrast, immunohistochemistry for CD34 would not show diffuse positivity; instead, fibroblasts are highlighted in a “fingerprint” pattern [1].

In dermatomyofibroma, extension into the superficial subcutis is rare. Further, elastic fibers are preserved or even slightly increased, which may be a helpful clue to the diagnosis. This tumor is also negative for CD34.

Plaque-like CD34-positive dermal fibroma, because of its positivity for CD34, as well as the presence of vague storiform patterns, may be misdiagnosed as DFSP. Plaque-like CD34-positive dermal fibroma is seen in the upper and mid-dermis, and a subepidermal grenz zone is also noted. Extension to the deep dermis and subcutis is rare in this tumor [1].

### 2.5. Sclerosing

Sclerosing DFSP is a term that was first proposed by Diaz-Cascajo and colleagues [23] in their report of two cases of DFSP, with large areas of the tumor embedded in a dense sclerotic stroma, although an earlier abstract by Kamino et al. described a series of 13 cases of a “collagenous” variant of DFSP in 1994, and a similar case of “sclerotic DFSP” was also described in an abstract by Barr et al. [24].

To be classified as sclerosing DFSP, ≥50% of the tumor must be embedded in a densely sclerotic and hypocellular stroma [23,25]. Areas of sclerosis are usually seen superficially and are not associated with any history of external trauma or previous/concurrent therapy, and, thus, have been suggested to be possibly due to tumor regression [23]. Different patterns of sclerosis have been described: parallel and concentric collagen bundles with clefting (sclerotic fibroma-like), homogenized collagen (lichen sclerosus or morphea-like), or a mixture of both patterns [25]. Thus, the differential diagnosis of this variant includes lichen sclerosus, morphea, sclerotic fibroma, and sclerosing epithelial fibrosarcoma. 

Lichen sclerosus and morphea would more often present clinically as white plaques and not as a mass, unlike most cases of DFSP. Lichen sclerosus also has a predilection for the anogenital area [26]. 

Sclerotic fibroma is characteristically well-circumscribed and polypoid [1,25]; however, in small biopsies, differentiation may be challenging on the basis of histomorphology alone. To further compound the difficulty, both entities are positive for CD34, and both have also shown variable positivity for EMA [25]. Thus, the definitive diagnosis is best rendered after excision. Molecular testing (such as FISH testing) to detect the characteristic gene arrangements of DFSP may also be conducted.

Sclerosing epithelial fibrosarcomas are often deep seated, unlike DFSP, which is usually more superficial in location. The sclerotic stroma of sclerosing epithelioid fibrosarcomas is associated with relatively monomorphic cells with epithelioid morphology, which is not characteristic of the tumor cells of DFSP. These tumors are strongly and diffusely positive for MUC4 in 80–90% of cases, with variable expression of EMA. Molecular testing for EWSR1-CREB3L1 rearrangement can also help confirm the diagnosis [8]. 

### 2.6. Granular Cell

Granular cell DFSP was first reported by Banerjee et al. in 1990 and has, so far, only been reported in four cases [27,28,29] in the literature. This variant is characterized by areas with larger polygonal cells, which have central ovoid nuclei, occasionally prominent nucleoli, and abundant granular eosinophilic cytoplasm. The granules in the cytoplasm of these cells also show diastase-resistant PAS positivity [27]. 

Ultrastructural studies show that these granular cytoplasmic changes are related to numerous autophagic or lysosome-like vacuoles [28]. Granular cell change is seen in other tumors, such as basal cell carcinoma, muscle tumors, neural tumors, and vascular tumors, and adequate sampling or excision to show the more classic spindle cell areas of DFSP is needed to rule these tumors out, as well as clinical correlation and immunohistochemical studies.

One important differential is granular cell tumor, which is a neuroectodermal tumor composed of similar-appearing granular cells. These tumors are positive for S100 and CD63 (NKI/C3), and strong nuclear TFE3 staining has also been demonstrated, [8] so immunohistochemistry can be used to exclude this entity. Perineural invasion and pseudoepitheliomatous hyperplasia is also common.

### 2.7. Fibrosarcomatous

**Figure 5:**
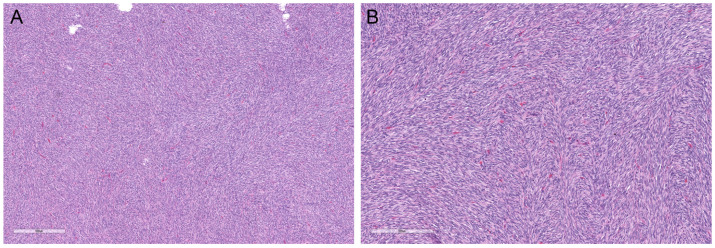
Fibrosarcomatous DFSP. Fibrosarcomatous transformation is characterized by areas showing increased cellularity and herringbone architecture, reminiscent of fibrosarcoma ((**A**); magnification ×40). This finding represents transformation to higher-grade tumor and carries a risk of metastasis. Mitotic rate can be increased ((**B**); magnification ×100) and necrosis can be occasionally present. However, these two features are not required for diagnosis.

Fibrosarcomatous DFSP was first described by Mentzel et al. in 1998 [30]. It is characterized by areas of herringbone or fascicular growth pattern, with increased cellularity, atypia, and mitotic activity (Figure 5A,B). The transition between areas of classic DFSP and areas of fibrosarcomatous DFSP is often abrupt.

CD34 positivity may be lost in fibrosarcomatous areas, which further complicates diagnosis, especially in small biopsy specimens. Adequate tissue sampling is, therefore, vital to make the diagnosis. For difficult cases, molecular testing for *COL1A1-PDGFB* rearrangement can be conducted. Some cases of the fibrosarcomatous variant also exhibit gains in genomic copies of *COL1A1-PDGFB* [31].

The differential diagnosis of fibrosarcomatous DFSP includes other spindle cell sarcomas, in which assessment of immunohistochemistry for other lineage-specific markers is useful. Adult fibrosarcoma is a diagnosis of exclusion, as it is, by definition, negative for CD34 and other markers. Adult fibrosarcoma can be distinguished from fibrosarcomatous DFSP on the basis of the depth of the tumor, as fibrosarcoma most often involves the deep soft tissues of the extremities, trunk, and head and neck.

Among all the variants of DFSP, fibrosarcomatous DFSP is the only variant that is clinically significant, as it is associated with an even higher risk of recurrence than classic DFSP and also has metastatic potential [32]. The most common site of distant metastasis of fibrosarcomatous DFSP is the lung [1]. A study by Erdem et al. [33] showed that fibrosarcomatous change was significantly associated with shorter recurrence-free survival in both univariate and multivariate analyses. Rarely, DFSP can also show sarcomatous transformation, exhibiting more pleomorphism and brisk mitotic activity, and these tumors may be mistaken for undifferentiated pleomorphic sarcoma [34]. 

## 3. Conclusions

These variants of DFSP, though rare, should still be part of the differential diagnosis of cutaneous spindle cell neoplasms, as errors in diagnosis may lead to over- or undertreatment of patients. Correlation with clinical data, especially appearance, location, depth, and borders, is helpful in the diagnosis of rare variants of DFSP, as are ancillary studies, such as immunohistochemistry and molecular testing. These help medical practitioners to avoid pitfalls in the diagnosis of these variants and ensure that patients are cared for correctly and adequately.
